# *N*‐Heterocyclic Carbene Stabilized Bisacylgermylenes

**DOI:** 10.1002/chem.202501707

**Published:** 2025-06-12

**Authors:** Matthias Paris, Roland C. Fischer, Anne‐Marie Kelterer, Michael Haas

**Affiliations:** ^1^ Institute of Inorganic Chemistry Graz University of Technology Stremayrgasse 9/IV Graz 8010 Austria; ^2^ Institute of Physical and Theoretical Chemistry Graz University of Technology Stremayrgasse 9/I Graz 8010 Austria

**Keywords:** acylgermanes, germanium, germylenes, N‐heterocyclic carbenes, subvalent compounds

## Abstract

In this contribution, a metal‐free synthetic approach toward isolable bisacylgermylenes, a novel class of germylenes, is described. Starting from tetra(2,4,6‐trimethylbenzoyl)germane **1** and bromo‐tris(2,4,6‐trimethylbenzoyl)germane **3**, we demonstrate that simple treatment with NHCs leads to two distinct types of reactivity: acyl abstraction and germylene stabilization. Reaction of **1** with NHCs produces imidazolium‐substituted germenolates **2a**,**b** via a hydrogen atom transfer (HAT) mechanism. In contrast, compound **3** undergoes stepwise substitution to afford isolable NHC‐stabilized bisacylgermylenes **4a**,**b**, which were structurally characterized by NMR and single‐crystal X‐ray diffraction. The UV/Vis spectra of **4a**,**b** show two absorption bands in the visible region of the light, which do not overlap and have different characters. Reactivity studies revealed its nucleophilic character and resulted in the formation of several germylene/iron and germylene/boron complexes. The removal of the NHC at room temperature with triphenylborane leads to a degradation, mainly based on the instability of the unstabilized bisacylgermylene. To determine its reactivity, an alternative synthetic strategy was developed based on the reaction of the geminal bisgermenolates **7** with the bischloro‐bis(2,4,6‐trimethylbenzoyl)‐germane **9**. Trapping reactions with NHCs results in the formation of **4a**,**b** in near quantitative yields. This work expands the synthetic scope of main group low‐valent chemistry and introduces germylenes with tunable reactivity.

## Graphical abstract

We developed a metal‐free strategy for acyl group abstraction, enabling access to novel germenolates **2a**,**b,** or NHC‐stabilized bisacylgermylenes **4a,b**. This novel class of germylenes exhibit nucleophilic reactivity toward iron and boron electrophiles, and can alternatively be prepared by the reaction of dichlorobisacylgermane and geminal bisgermenolate at low temperature.

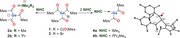

## 1 Introduction

**Scheme 1:**
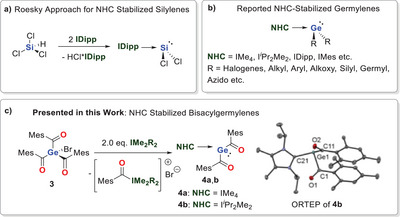
a) Roesky approach for NHC‐ stabilized silylenes, b) reported NHC‐stabilzed germylenes, c) novel NHC‐ stabilized germylenes presented in this work.

The synthesis of low‐valent compounds featuring heavier group 14 elements is one of the focal points of modern main group chemistry.^[^ ^1^ ^]^ Today, it is broadly recognized that low valent main group elements play a key role in synthesis, catalysis, and small molecule activation.^[^ ^2^ ^]^ Among them, heavier carbene analogues and their dimers (stabilized or not) are of particular interest. A landmark paper, in this respect, is the metal‐free reductive elimination of HCl from trichlorosilane induced by a *N*‐heterocyclic carbene toward a NHC‐ stabilized silylene (see Scheme 1a).^[^ ^3^ ^]^ This approach has since inspired numerous studies, significantly expanding the library of isolable silylenes.^[^ ^4^ ^]^

For germanium analogues, various synthetic routes to NHC‐stabilized germylenes have been developed.^[^ ^5^ ^]^ The pioneer of this chemistry was Arduengo et al. by the isolation of a diiodogermylene stabilized by a NHC.^[^ ^6^ ^]^ Since then a variety of different substituents at the germanium atom have been reported (see Scheme 1b).^[^ ^7^, ^8^, ^9^ ^]^

Recently, we reported on a series of electron transfer reactions of acylgermanes toward radical anions,^[^ ^10^ ^]^ germenolates,^[^ ^11^ ^]^ as well as geminal bisgermenolates.^[^ ^12^ ^]^ All these reactions were either induced by alkali metals or by alkali metal alkoxylates. Motivated by these findings, we asked whether mild, metal‐free conditions could unlock similar transformations offering cleaner access to sensitive main‐group species without the pitfalls of metal‐based methods. Given the high Brønsted basicity of NHCs such as 1,3‐diisopropyl‐4,5‐dimethylimidazol‐2‐ylidene (I*^i^* Pr_2_Me_2_, pKa ≈ 24 in DMSO),^[^ ^13^ ^]^ we set out and reacted two selected examples of acylgermanes (tetra(2,4,6‐trimethylbenzoyl)germane **1** and bromo‐tris(2,4,6‐trimethyl‐benzoyl)germane **3**) with I*^i^* Pr_2_Me_2_ and 1,3‐dimethyl‐4,5‐dimethylimidazol‐2‐ylidene (IMe_4_). We found that acylgermanes can engage in diverse and highly selective transformations with NHCs, enabling direct access to germenolates and germylenes (see Scheme 1c) without requiring external reductants or metal catalysts.

## 2 Results and Discussion

Tetraacylgermane **1** was chosen as starting material due to its known redox activity,^[^ ^10^ ^]^ offering a promising platform for selective transformations at the germanium center.

### 2.1 Synthesis of Metal‐Free Germenolates

**Scheme 2:**
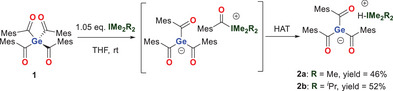
Generation of the germenolates **2a** and **2b** by a NHC induced acyl abstraction starting from **1**.

To our delight, the reaction of tetra(2,4,6‐trimethylbenzoyl)‐germane (**1**) with NHCs such as I*^i^* Pr_2_Me_2_ and IMe₄ resulted in a clean, selective abstraction of one mesitoyl group. Interestingly, the initial formed germenolate undergoes a hydrogen atom transfer (HAT) where the initially formed mesitoyl group of the azolium ion is replaced by a hydrogen atom (see Scheme 2). A similar reactivity of acyl azolium derivatives were found by Gutierrez, Scheidt, and coworkers.^[^ ^14^ ^]^

After precipitation with *n*‐pentane the germenolates **2a** and **2b** were isolated as orange crystalline solids in good yields. These metal‐free germenolates were characterized by ^1^H‐ and ^13^C‐ NMR spectroscopy, using C_6_D_6_ as solvent. The most notable ^13^C NMR spectroscopic property of **2a**,**b** are the considerably downfield‐shifted signals for the carbonyl C‐atom for **2a** at *δ* = 261.9 ppm and for **2b** at *δ* = 260.9 ppm, which is characteristic for carbonyl groups directly linked to a negative charged germanium atom.^[^ ^15^ ^]^ In contrast to this, the former carbenic carbons undergo a significant upfield shift toward *δ* = 148.64 ppm for **2a** and *δ*  =  148.83 ppm for **2b**, supporting the transformation of the NHC into an aromatic azolium ion. In addition, the abstracted proton show also significantly downfield shifted ^1^H chemical signals for **2a** at *δ*  =  10.28 ppm and for **2b** at *δ* = 11.60 ppm, making it a distinct feature of the protonated imidazolium ion.^[^ ^16^ ^]^ All other analytical data are consistent with the proposed structures and are summarized in the Supporting Information (see Figure S1–S4).

**Figure 1:**
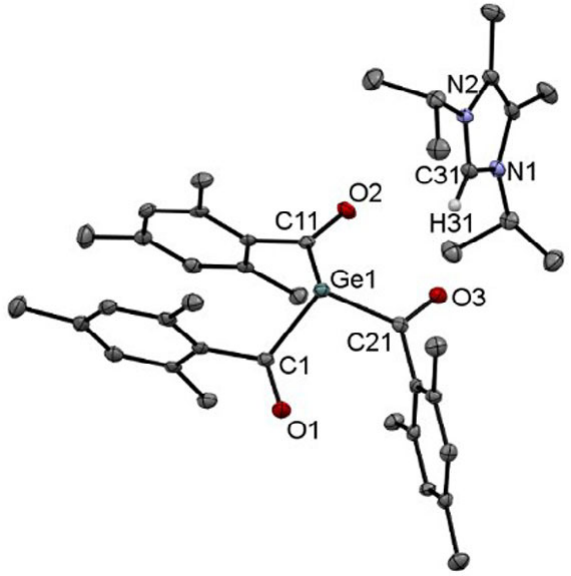
ORTEP representation for compound **2b**. Thermal ellipsoids are depicted at the 50% probability level. Hydrogen atoms except the Hydrogen of the imidazolium moiety are omitted for clarity. Selected bond lengths (Å) and bond angles (deg) with estimated standard deviations: ΣαGe(1) 309.26, N(1)─C(31)─N(2) 108.45(16), Ge(1)─C(1) 2.0307(18), Ge(1)─C(11) 2.0087(19), Ge(1)─C(21) 2.0200(18), C(1)─O(1) 1.223(2), C(11)─O(2) 1.234(1), C(21)─O(3) 1.236(2), C(31)─N(1) 1.333(2), C(31)─N(2) 1.332(2).

Upon cooling a concentrated Et₂O solution of derivative **2b** to − 30 °C, crystals suitable for single‐crystal X‐ray analysis were formed. The molecular structure is depicted in Figure 1. Compound **2b** crystallizes in the triclinic space group *P*‐1 containing two molecules per unit cell.

### 2.2 Synthesis of NHC‐Stabilized Bisacylgermylenes

**Scheme 3:**
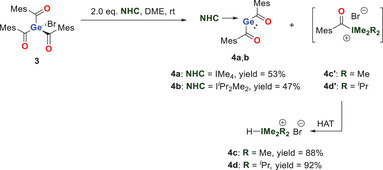
Generation of the NHC‐stabilized bisacylgermylenes **4a** and **4b**.

This straightforward NHC‐mediated acyl abstraction served as a strategic entry point toward the synthesis of NHC‐stabilized tetrylenes (see Scheme 1c). We envisioned bromo‐tris(2,4,6‐trimethylbenzoyl)germane **3** as an ideal precursor molecule for this research question.^[^ ^17^, ^18^ ^]^ Therefore, we reacted **3** with two equivalents of IMe_4_ and I*^i^* Pr_2_Me_2_ at room temperature. The first equivalent of the NHC removes one mesitoyl group and the bromide generating the corresponding salt. Instead of the acyl azoliums **4c’** and **4d’**, we isolated the protonated species **4c** and **4d**, which are generated via a HAT pathway in excellent yields. The second equivalent of NHC stabilizes the resulting germylene and thus forming the base stabilized germylenes. After recrystallization in DME at − 30 °C we isolated **4a** and **4b** in yields of 53% and 47%, respectively, as orange crystalline solids (see Scheme 3). **4a**,**b** were characterized by ^1^H and ^13^C NMR spectroscopy with C_6_D_6_ as solvent. In the ¹^3^C NMR spectrum, the carbonyl signals were observed at *δ* = 260.58 ppm for **4a** and 259.35 ppm for **4b**, both significantly downfield‐shifted compared to those of acylgermanes. However, the carbonyl shifts are in the region typically for germenolates, which indicates the presence of a lone pair at the germanium atom.^[^ ^15^ ^]^

In addition, the carbenic carbons undergo a significant high field shift [*δ* = 166.70 for IMe_4_ and *δ* = 167.67 ppm for I*^i^* Pr_2_Me_2_] compared to the corresponding free NHCs, confirming the coordination to the germanium center.^[^ ^6^, ^8^, ^19^, ^20^ ^]^ Interestingly, a significant deshielding of the ^1^H shift of the CH─(CH_3_)_2_ attached to the isopropyl groups in **4b** was observed [*δ* = 5.81 ppm, compared to the signal for the free I^i^Pr_2_Me_2_ at *δ* = 3.95 ppm]. This shift toward lower field further confirms the attachment of the NHC and is consistent with observations in similar NHC‐ stabilized germylenes.^[^ ^7^, ^8^, ^19^, ^20^ ^]^

The protonated species **4c** and **4d** were also characterized by ^1^H and ^13^C NMR spectroscopy with CDCl_3_ as solvent. The imidazolium proton resonates at *δ* = 9.70 ppm (**4c**) and *δ* = 10.30 ppm (**4d**), consistent with azolium formation. Full assignments are given in the Supporting Information (see Figure S5–S12).

**Figure 2:**
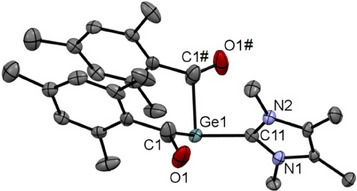
ORTEP representation of **4a**. Thermal ellipsoids are depicted at 50% probability level. Selected bond lengths (Å) and bond angles (deg): ΣαGe(1) 288.74, N(1)─C(11)─N(2) 105.0(16), Ge(1)─C(1) 2.131(7), Ge(1)─C(11) 2.039(17), C(1)─O(1) 1.160(8), C(11)─N(1) 1.313(17), C(11)─N(2) 1.348(17).

**Figure 3:**
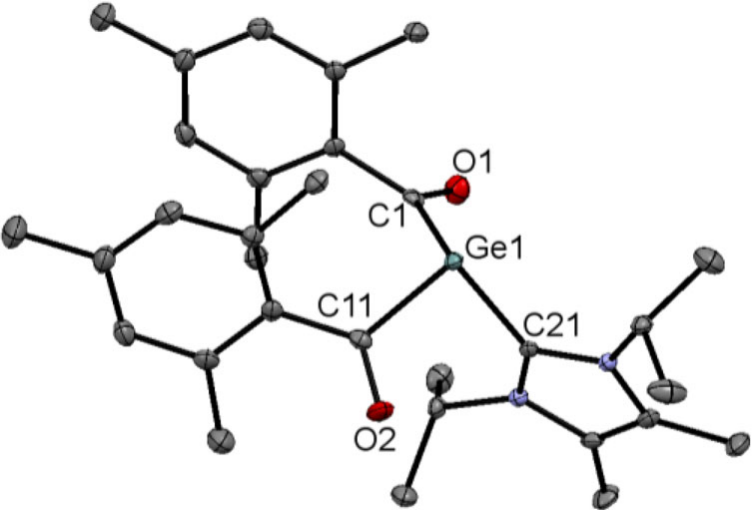
ORTEP representation of **4b**. Thermal ellipsoids are depicted at 50% probability level. Selected bond lengths (Å) and bond angles (deg): ∑αGe(1) 306.28, C(1)─Ge(1)─C(11) 108.06(11), C(1)─Ge(1)─C(21) 100.80(11), C(11)─Ge(1)─C(21) 97.42(11), Ge(1)─C(1) 2.025(3), Ge(1)─C(11) 2.034(3), Ge(1)─C(21) 2.050(3), C(1)─O(1) 1.221(3), C(11)─O(2) 1.224(4).

Single crystals of **4a,b** suitable for X‐ray structural analysis could be grown by cooling a concentrated solution of **4a,b** in DME to − 30 °C. The molecular structures are depicted in Figures 2 and 3. An interesting structural feature for both compounds is that the mesityl groups all parallel to each other indicating strong π stacking. Consequently, the distance between the aromatic groups are significant smaller compared to the tetra(2,4,6‐trimethylbenzoyl)‐germane (**1**) [0.17 Å for **4a** and by 0.10 Å for **4b**].^[^ ^21^ ^]^ While compound **4a** shows significantly elongated Ge─C single bonds, **4b** adopts only slightly elongated Ge─C bonds. For both compounds the Ge1─C─carbenic [2.039(17) Å for **4a** and 2.050(3) Å for **4b**] are slightly shorter than those of the dimesitylgermylene‐NHC complexes with IMe_4_ (2.067(3) Å) and I*^i^* Pr_2_Me_2_ (2.078(3)  Å).^[^ ^8^ ^]^

**Figure 4:**
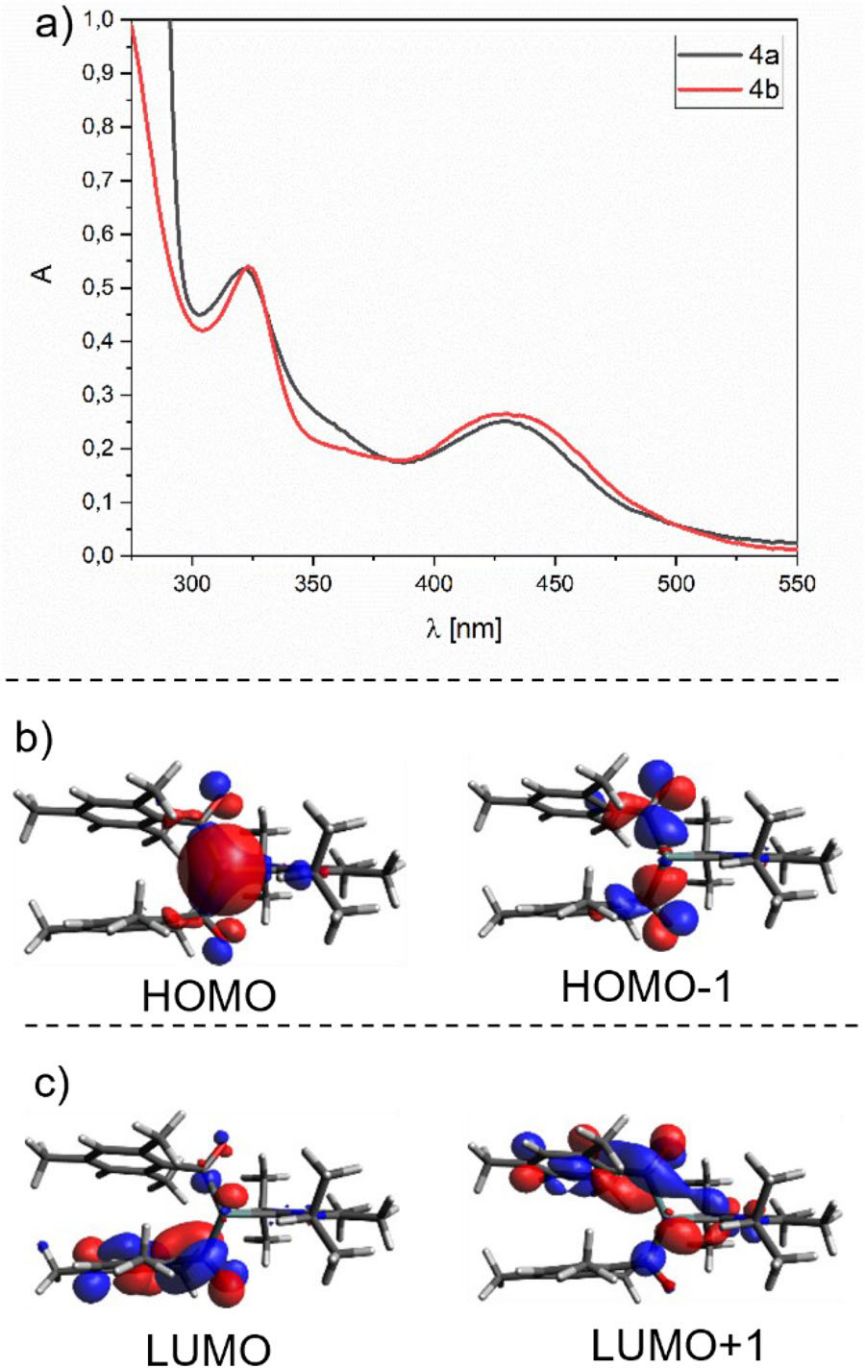
a) Experimental spectra of **4a**,**b** in THF at 1× 10^−4 ^mol/L, b) HOMO orbitals involved in the two transitions constituting the first absorption band. c) LUMO orbitals involved in the two transitions constituting the first absorption band. For details see the Supporting Information Tables S2–S5.

The UV/Vis spectra of **4a**,**b** showed two characteristic bands in the visible region of light at *λ* = 322, and 430 nm, respectively (Figure 4a). Time‐dependent DFT (TD‐DFT) calculations reveal that the bands at *λ* = 322 nm correspond to the n→π* transition from the carbonyl‐oxygen lone pairs toward the π system including the mesitoyl and NHC moieties. The lower‐energy bands at *λ* = 430 nm are attributed to two excitations from the n orbitals and the Ge‐centered lone pair orbitals to the mesitoyl π* system (Figure 4b,c).

Germylenes **4a**,**b** exhibit notably low electrophilic reactivity, as demonstrated by their lack of reactivity toward a broad range of unsaturated hydrocarbons—including phenylacetylene and 2,3‐dimethylbutadiene—as well as common silanes such as Ph₃SiH, Et₃SiH, and Ph₂SiH₂. This pronounced inertness suggests that both compounds are electronically stabilized or sterically protected, limiting their ability to undergo typical electrophilic additions. The observed stability may arise from effective delocalization of the lone pair at the germanium center or an energetically inaccessible LUMO, making these germylenes unreactive under standard conditions.

**Scheme 4:**
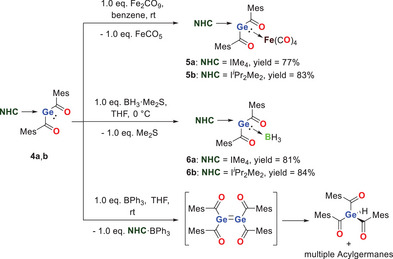
Performed reactions to evaluate the donor acceptor ability of **4a**,**b**.

The donor acceptor ability of **4a**,**b** was evaluated by the reaction with Fe_2_CO_9_ and BH_3_•SMe_2_ (Scheme 4). Numerous transition metal complexes are already known for base‐coordinated germylenes.^[^ ^22^ ^]^ In addition, the coordination of the BH_3_ moiety is also well established as benchmark for nucleophilicity.

In both cases the germylenes were added at room temperature in excellent yields to the Fe(CO)_4_ group. **5a**,**b** were characterized by ^1^H and ^13^C NMR spectroscopy with THF‐d_8_, and C_6_D_6_, respectively, as solvent. Based on the coordination of the NHC‐ stabilized germylene to the iron moiety, the carbonyl groups at the germanium undergo a significant high field shift in comparison to **4a**,**b**. As expected the carbonyl groups at the iron center display a significant downfield shift. This is well in line with the literature.^[^ ^23^ ^]^ All other resonances are also assigned and can be found in the Supporting Information Figures S13–S16.

**Figure 5:**
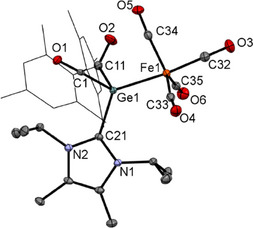
ORTEP representation for compound **5b**. Thermal ellipsoids are depicted at the 50% probability level. Hydrogen atoms are omitted and mesityl groups are displayed as wireframes for clarity. Selected bond lengths (Å) and bond angles (deg) with estimated standard deviations: ΣαGe(1) 308.18, Ge(1)─C(1) 2.0633(15), Ge(1)─C(11) 2.0639(16), Ge(1)─C(21) 2.0553(16), Ge(1)─Fe(1) 2.4052(3), C(21)─N(1) 1.3548(19), C(21)─N(2) 1.363(2), C(1)─O(1) 1.2172(19), C(11)─O(2) 1.213(2), Fe(1)─C(32) 1.7719(18), Fe(1)─C(33) 1.7827(18), Fe(1)─C(34) 1.7932(18), Fe(1)─C(35) 1.7836(17), C(32)─O(3) 1.151(2), C(33)─O(4)1.155(2), C(34)─O(5) 1.150(2), C(35)─O(6) 1.153(2).

Single crystals of **5a,b** suitable for X‐ray structural analysis could be grown by cooling a concentrated solution of **5a,b** in a mixture of toluene:THF 3:2 to − 30 °C. The molecular structure of **5b** as representative example is depicted in Figure 5. The molecular structure of **5a** can be found in Figure S30. Both derivatives show similar coordination geometry with bond distances and angles that differ only slightly. Moreover, the germanium atoms are tetracoordinated with a distorted tetrahedral geometry. The Fe─Ge bond lengths in **5a**,**b** (2.3879(4) for **5a** and 2.4052(3) for **5b**) are slightly elongated compared to the literature known values for tetracoordinated germylene iron carbonyl complexes (2.298–2.348°A).^[^ ^24^ ^]^ We assume that this effect is caused by steric congestion.

We also reacted the germylene **4a,b** with BH_3_·Me_2_S to generate the adducts **6a**,**b** which were isolated as yellow solids in high yields of 80% for **6a** and 84% for **6b**. **6a**,**b** were characterized by ^1^H, ^11^B, and ^13^C NMR spectroscopy with C_6_D_6_ as solvent (Figures S17‐S24 in the Supporting Information).

Again, the coordination of the boron moiety induces a significant high field shift of the carbonyl groups, in comparison to **4a**,**b**. The presence of boron in the products was established by ^11^B NMR spectroscopy, which yielded a broad resonance at *δ* = −35 ppm for **6a** and at *δ* = −34 ppm for **6b**, corresponding to a coordinated BH_3_ group. Interestingly, the BH_3_ protons were not resolved in the ^1^H NMR due to broadening, but IR spectroscopy revealed characteristic B─H stretching vibrations at 2200–2300 cm^−1^, further supporting the presence of the BH_3_ moiety and strengthening the overall structural characterization.^[^ ^9^, ^20^ ^]^

**Figure 6:**
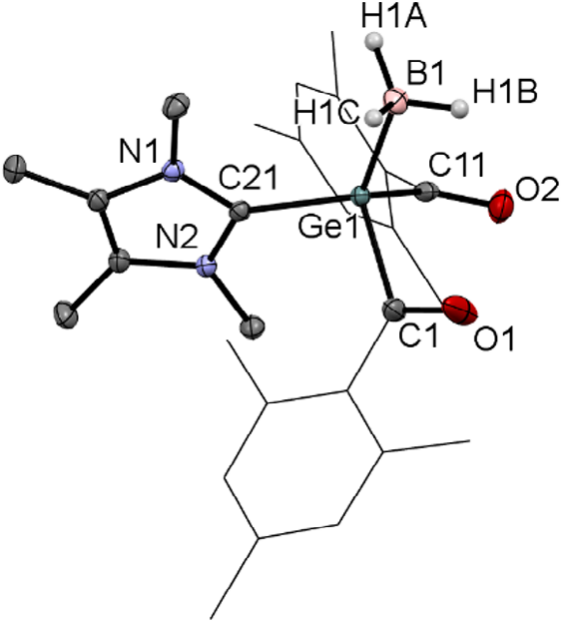
ORTEP representation for compound **6a**. Thermal ellipsoids are depicted at the 50% probability level. Hydrogen atoms are mostly omitted and mesityl groups are displayed as wireframes for clarity. Selected bond lengths (Å) and bond angles (deg) with estimated standard deviations: ΣαGe(1) 311.63, Ge(1)─C(1) 2.0316(18), Ge(1)─C(11) 2.0329(18), Ge(1)─C(21) 2.0095(18), Ge(1)─B(1) 2.062(2), C(21)─N(1) 1.352(2), C(21)─N(2) 1.352(2), C(1)─O(1) 1.216(2), C(11)─O(2) 1.216(3).

Single crystals of **6a** suitable for X‐ray structural analysis could be grown by cooling a concentrated solution of **6a** in THF to − 30 °C. The molecular structure of **6a** is depicted in Figure 6. The Ge − B bond length is 2.062(2) Å, which is slightly shorter than that reported for Baine's germylene─BH₃ adduct.^[^ ^20^ ^]^ However, it is comparable to other germylene─BH₃ complexes, which typically fall within the range of 2.016 to 2.053 Å.^[^ ^9^, ^25^ ^]^ In order to prepare the NHC free germylene, which should dimerize to the corresponding tetrakisacyldigermene, we used the sterically encumbered Lewis acid BPh_3_.^[^ ^26^ ^]^ While the reaction proved to form the adduct NHC·BPh_3_, on the basis of NMR analysis, the unstabilized germylene or the digermene was not observed. Instead a variety of different acylgermanes, with trisacylgermane being the main product, were formed (see in Figure S25 for the crude NMR after the reaction). Consequently, we assume, that without the stabilization of a donor molecule, this low valent compound is not stable at room temperature. This observation is in line with a previous report by Scheschkewitz and coworkers on transient acyl digermenes.^[^ ^27^ ^]^

Nevertheless, we explored an alternative pathway to prepare the tetrakisacyldigermene at lower temperature, aiming to trap this elusive species. To access the digermene, we selected the geminal bisgermenolate **7** (previously synthesized by our group) as the starting material.^[^ ^12^ ^]^ The only remaining precursor was the corresponding dihalogermanium compound, which we synthesized as described below.

As starting point for our pathway we again used the bisgermenolate **7**. Compound **7** was added as a solid to an excess of HCl dissolved in Et_2_O at − 70 °C. After removal of all volatiles, the corresponding dihydride **8** was then isolated in near quantitative yields of 96% as a yellow oil. By cooling a concentrated solution of **8** in *n*‐pentane to − 30 °C we were able to grow single crystals suitable for X‐ray analysis (see Figure S31).

The synthesis of bischloro‐bisacyl‐germane **9** was achieved by refluxing the dihydride **8** in an excess of CCl_4_. Complete chlorination was monitored via NMR and the product was isolated after removal of all volatiles in near quantitative yields of 96% as a yellow solid. By cooling a concentrated *n*‐pentane solution of **9** to − 30 °C and slowly evaporating it, we were able to grow single crystals suitable for X‐ray analysis (see Figure S32).

**Scheme 5:**
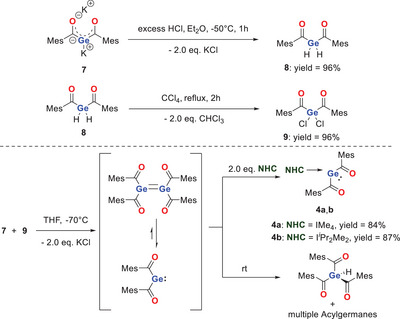
Synthetic strategy toward the unstabilized bisacylgermylene.

To investigate the reactivity of the germanium‐containing building blocks, we carried out a reaction between bisgermenolate **7** and dihalide **9** in THF at −70 °C. Subsequently, the reaction solution was allowed to warm up to room temperature. During this process the reaction mixture exhibited a noticeable color change, indicative of digermene formation. However, instead of forming the expected digermene, the reaction led to a rearrangement process, resulting again in the formation of several acyl‐substituted germanium compounds. NMR spectroscopy revealed a complex product mixture, with the trisacylgermane identified as the major product. To probe the formation of low‐valent germanium species in this transformation, we performed trapping experiments using I*^i^* Pr_2_Me_2_ and IMe₄. In both cases, the reactions led to the clean formation of the corresponding NHC‐stabilized bisacylgermylenes **4a**,**b** in excellent yields. These results strongly support the transient generation of low‐valent germanium intermediates by this approach (Scheme 5).

## 3 Conclusion

In conclusion, we have established a selective and metal‐free abstraction pathway for acyl groups. The reaction of NHCs with tetraacylgermane **1** results in the formation of imidazolium substituted germenolates **2a**,**b**. Moreover, the reaction of the same NHCs with bromo‐trisacylgermane **3** results in the formation of NHC‐ stabilized bisacylgermylenes **4a**,**b** as novel class of germylenes. It highlights the potential of NHCs not only as ligands but as active reagents capable of driving stoichiometric transformations at heavier p‐block elements. The observed HAT pathway is particularly noteworthy and may find analogs in related main‐group or organocatalytic systems. In addition, we performed reactivity studies on **4a** and **4b**, demonstrating their nucleophilic character, leading to the formation of various germylene/iron (**5a**,**b**) and germylene/boron (**6a**,**b**) complexes. When the NHC is removed at room temperature using triphenylborane, degradation occurs, primarily due to the instability of the unstabilized bisacylgermylene. To assess its reactivity, an alternative synthetic approach was developed involving the reaction of geminal bisgermenolates **7** with bischloro‐bisacyl‐germane **9**. Trapping reactions at −70 °C with NHCs produce compounds **4a** and **4b** in nearly quantitative yields. Further studies to probe the scope of this chemistry are currently in progress.

## 4 Experimental Section

### Synthesis

All experiments were performed under a nitrogen atmosphere using standard Schlenk techniques. Solvents were dried using a column solvent purification system.^[^ ^28^ ^]^ Me_3_SiCl (≥ 99%), GeCl_4_ (99,99%), KO*t*Bu (> 98%), THF‐d_8_ (99.5 atom% D), chloroform‐d (99.8 atom%, D), and benzene‐d_6_ (99.5 atom%, D) were used without any further purification. For the measurement of air‐sensitive samples, benzene‐d_6,_ and THF‐d_8_ were additionally dried above a sodium/potassium alloy at 12‐hour reflux. Also, chloroform‐d was dried with type 4A molecular sieves. Tetrakis(trimethylsilyl)germane,^[^ ^29^ ^]^ tetraacylgermane **1,** and FC(O)Mes was prepared according to published procedures.^[^ ^21^ ^]^ Bromo‐tris(2,4,6‐trimethylbenzoyl)germane **3** was prepared according to the recently published procedure.^[^ ^18^ ^]^ Melting points were determined using the Stuart SMP50 apparatus and are uncorrected. Elemental analyses were carried out on a Hanau Vario Elementar EL apparatus.

### UV/Vis Spectroscopy

UV/Vis spectra were acquired either using a TIDAS UV/Vis spectrometer equipped with optical fibers and a 1024‐pixel diode‐array detector (J&M Analytik AG, Essingen, Germany), or a Perkin Elmer Lambda 5 spectrometer.

### FT‐IR Spectroscopy

Infrared spectra were obtained on a Bruker α‐P Diamond ATR Spectrometer running OPUS 7.5 software in transmission mode from the solid sample.

### NMR Spectroscopy

^1^H, and ^13^C NMR spectra were recorded either on a Varian INOVA 400, a 200 MHz Bruker AVANCE DPX, or a 400 MHz spectrometer Jeol JNM‐ECZL with Royal HFX‐Probes with auto sampler and referenced versus TMS using the internal ^2^H‐lock signal of the solvent.

### X‐Ray Crystallography

For single crystal X‐ray diffractometry all suitable crystals were covered with a layer of silicone oil. A single crystal was selected, mounted on a glass rod on a copper pin, and placed in the cold N_2_ stream provided by an Oxford Cryosystems cryometer (T = 100 K), if not otherwise stated. XRD data collections for all structures reported were performed on a Bruker APEX II diffractometer with use of Mo Kα radiation (*λ* = 0.71073 Å) from an IµS microsource and a CCD area detector. Empirical absorption corrections were applied using SADABS.^[^ ^30^ ^]^ The structures were solved with use of either direct methods or the Patterson option in SHELXS. Structure refinement was carried out using SHELXL.^[^ ^31^ ^]^ CIF files were edited, validated, and formatted with the program OLEX2.^[^ ^32^ ^]^ The space group assignments and structural solutions were evaluated using PLATON.^[^ ^33^ ^]^ All nonhydrogen atoms were refined anisotropically. All hydrogen atoms were placed in calculated positions corresponding to standard bond lengths and angles using riding models. CCDC deposition numbers 2 374 269–2374276 contain the supplementary crystallographic data for compound in this paper. These data can be obtained free of charge via www.ccdc.cam.ac.uk/data_request/cif.

### Density Functional Theory (DFT) Calculations

Starting from the X‐ray structures, the geometries of compounds **4a** and **4b** were optimized by DFT using the composite method PBEh‐3c,^[^ ^34^ ^]^ which uses the m‐def2‐SVP basis set.^[^ ^35^ ^]^ PBEh‐3c applies the PBE0 hybrid functional with geometrical counterpoise correction accounting for the basisset superposition error and Grimme's D3BJ dispersion correction including Becke‐Johnson damping. Frequencies in the Harmonic oscillator approximation were computed to confirm that the optimized geometries are minima at the potential energy surface. For the optimized minima, TD‐DFT was applied with the same method for the calculation of 15 vertical excitations. All calculations were performed in the conductor like polarizable continuum solvation model CPCM^[^ ^36^, ^37^ ^]^ in the solvent THF. All calculations were performed with the program ORCA, version5 using standard parameters.^[^ ^37^ ^]^

From the vertical excitation data, the simulation of the UV/Vis spectra was generated using the program orca_asa^[^ ^38^ ^]^ with a Gaussian broadening parameter sigma of 1500 cm^−1^. The resulting absorption bands were red‐shifted by 3500 cm^−1^ to account for the systematic shifts of the UV spectra as found by PBEh‐3c.

### 4.1 Experimental Procedures

**Synthesis of 2a**: 62 mg of 1,3‐dimethyl‐4,5‐dimethylimidazol‐2‐ylidene (IMe_4_) (0.50 mmol; 1.10 eq.) was dissolved in 4 mL of THF and slowly added to 300 mg of **1** (0.45 mmol; 1.00 eq.) dissolved in 10 mL THF at −80 °C. The reaction solution was brought to room temperature and stirred for 72 hours to ensure complete conversion of the starting material. After removal of the solvent and resuspending the crude product in 10 mL of toluene, the solution was cooled to −80 °C to ensure precipitation. The germenolate **2a** was isolated as an orange crystalline solid after filtration. **Yield**: 133 mg (0.21 mmol; 46%) of analytically pure **2a** as orange crystalline solid.

**mp**: 71–73 °C. **Anal. Calc**. (%) for C_37_H_46_GeN_2_O_3_: C, 69.50; H, 7.25, Found: C, 69.22; H, 7.44. **^13^C‐NMR** Data (benzene‐d_6_, TMS, ppm): 261.82 (*C *= O), 148.64 (N─*C*─N of IMe_4_), 138.21, 134.90, 131.50, 128.37 (Mes─*C*), 125.72 (*C*─CH_3_ of IMe_4_), 32.78 (N─*C*H_3_ of IMe_4_), 21.29 (Aryl─*C*H_3_), 20.48 (Aryl─*C*H_3_), 7.17 (C─*C*H_3_ of IMe_4_). **^1^H‐NMR** Data (benzene‐d_6_, TMS, ppm): 10.28 (s, 1H, N─C*H*─N of IMe_4_), 6.58 (s, 6H, Mes─*H*), 3.20 (s, 6H, N─C*H_3_* of IMe_4_), 2.47 (s, 18H, Mes─C*H*_3_), 2.19 (s, 9H, Mes─C*H*_3_), 1.13 (s, 6H, NC─C*H*_3_ of IMe_4_). **UV/Vis**: *λ* [nm] (*ε* [L mol^−1^ cm^−1^]) = 424 (4210), 350 (4694). **IR** (neat): *ν*(C = O) = 1639.

**Synthesis of 2b**: 90 mg of 1,3‐diisopropyl‐4,5‐dimethylimidazol‐2‐ylidene (I*^i^* Pr_2_Me_2_) (0.50 mmol; 1.10 eq.) was dissolved in 4 mL of THF and slowly added to 300 mg of **1** (0.45 mmol; 1.00 eq.) dissolved in 10 mL of THF at −80 °C. The reaction solution was brought to room temperature and stirred for 72 hours to ensure complete conversion of the starting material. After removal of the solvent and resuspending the crude product in 10 mL of toluene, the solution was cooled to −80 °C. Subsequently, this solution was added at −80 °C to 20 mL of n‐pentane to ensure precipitation. After filtration the product **1b** was isolated as an orange crystalline solid. **Yield**: 164 mg (0.24 mmol, 52%) of analytically pure **2b** as orange crystalline solid.

**mp**: 83–85 °C. **Anal. Calc**. (%) for C_41_H_54_GeN_2_O_3_: C, 70.80; H, 7.83, Found: C, 70.55; H, 7.93. **^13^C‐NMR** Data (benzene‐d_6_, TMS, ppm): 260.91 (*C *= O), 148.83 (N─*C*─N of I*^i^* Pr_2_Me_2_), 134.64, 131.50, 131.52, 128.30 (Mes─*C*), 125.27 (*C*─CH_3_ of I*^i^* Pr_2_Me_2_), 50.80 (N─*C*HCH_3_ of I*^i^* Pr_2_Me_2_), 22.34 (NCH─*C*H_3_ of I*^i^* Pr_2_Me_2_), 21.31 (Aryl─*C*H_3_), 20.52 (Aryl─*C*H_3_), 7.98 (NC─*C*H_3_ of I*^i^* Pr_2_Me_2_). **^1^H‐NMR** Data (benzene‐d_6_, TMS, ppm): 11.60 (s, 1H, NC*H*N of I*^i^* Pr_2_Me_2_), 6.61 (s, 6H, Mes─*H*), 3.83 (sept, J = 20.6 Hz, 6H, N─C*H* of I*^i^* Pr_2_Me_2_), 2.55 (s, 18H, Mes─C*H*_3_), 2.20 (s, 9H, Mes─C*H*_3_), 1.41 (d, J = 6.7 Hz, 12H, NCH─(C*H*_3_)_2_ of I*^i^* Pr_2_Me_2_), 1.32 (s, 6H, NC─C*H*_3_ of I*^i^* Pr_2_Me_2_). **UV/Vis**: *λ* [nm] (*ε* [L mol^−1^ cm^−1^]) = 426 (4538), 353 (4247). **IR** (neat): *ν*(C = O) = 1656, 1629, 1609.

**Synthesis of 4a and 4c**: 300 mg of **3** (0.50 mmol; 1.00 eq.) and 132 mg of IMe_4_ (1.06 mmol; 2.10 eq.) were combined in a flask. Subsequently, 15 mL of DME was added and stirred over night at room temperature. After complete conversion, the solvent was removed and washed with 15 mL of *n*‐pentane (the filtrate was discarded). The solid was washed three times with 10 mL of THF until only the white solid **4c** was left. After removal of the THF the resulting residue is recrystallized in DME at −30 °C and the product **4a** was isolated as an orange crystalline solid. **Yield**: 132 mg (0.27 mmol; 53%) of analytically pure **4a** as orange crystalline solid.

**Analytical data for 4a**: **mp**: 185–187 °C. **Anal. Calc**. (%) for C_27_H_34_GeN_2_O_2_: C, 66.02; H, 6.98, Found: C, 66.33; H, 7.21. **^13^C‐NMR** Data (benzene‐d_6_, TMS, ppm): 260.58 (*C *= O), 166.70 (N─*C*─N of IMe_4_), 146.71, 136.38, 132.28, 129.10 (Mes─*C*), 125.59 (*C*─CH_3_ of IMe_4_), 30.04 (N─*C*H_3_ of IMe_4_), 21.17 (Aryl─*C*H_3_), 8.25 (C─*C*H_3_ of IMe_4_). **^1^H‐NMR** Data (benzene‐d_6_, TMS, ppm): 6.58 (s, 4H, Mes─*H*), 3.34 (s, 6H, of IMe_4_), 2.52 (s, 12H, Mes─C*H*_3_), 2.16 (s, 6H, Mes─C*H*_3_), 1.20 (s, 6H, C─C*H*_3_ of IMe_4_). **UV/Vis**: *λ* [nm] (*ε* [L mol^−1^ cm^−1^]) = 429 (2508), 321 (5355). **IR** (neat): *ν*(C = O) 1605, 1639, 1650.

**Analytical data for 4c**: **Yield**: 91 mg (0.44 mmol; 88%) of analytically pure **4c** as white crystalline solid.

**mp**: 185–187 °C. **Anal. Calc**. (%) for C_7_H_13_BrN_2_: C, 40.99; H, 6.39, Found: C, 41.20; H, 6.44. **^13^C‐NMR** Data (CDCl_3_, TMS, ppm): 135.21 (N─*C*─N of IMe_4_), 127.08 (*C*─CH_3_ of IMe_4_), 34.25 (N─*C*H_3_ of IMe_4_), 8.76 (C─*C*H_3_ of IMe_4_). **^1^H‐NMR** Data (CDCl_3_, TMS, ppm): 9.70 (s, 1H, *H*─IMe_4_), 3.80 (s, 6H, of IMe_4_), 2.19 (s, 6H, C─C*H*_3_ of IMe_4_).

**Synthesis of 4b and 4d**: 400 mg of **3** (0.67 mmol; 1.00 eq.) and 255 mg of I*^i^* Pr_2_Me_2_ (1.41 mmol; 2.10 eq.) were combined in a flask, 15 mL *n*‐pentane was added and stirred over night at room temperature. Then the reaction solution was filtrated, washed with *n*‐pentane, and the filtrate was discarded. The solid was washed with THF until only the white solid **4d** was left. After removal of the solvent the resulting residue was dissolved in 10 mL of DME, recrystallized at −30 °C, and the product **4b** was isolated as an orange crystalline solid. **Yield**: 174 mg (0.32 mmol; 47%) of analytically pure **4b** as orange crystalline solid.

**Analytical data for 4b**: **mp**: 167–169 °C. **Anal. Calc**. (%) for C_31_H_42_GeN_2_O_2_: C, 68.03; H, 7.74, Found: C, 68.34; H, 7.92. **^13^C‐NMR** Data (benzene‐d_6_, TMS, ppm): 259.35 (*C *= O), 167.67 (N─*C*─N of I*^i^* Pr_2_Me_2_), 146.51, 136.21, 132.13, 129.05 (Mes─*C*), 126.62 (*C*─CH_3_ of I*^i^* Pr_2_Me_2_), 53.70 (N─*C*H─CH_3_ of I*^i^* Pr_2_Me_2_) 21.88 (N─*C*H_3_ of I*^i^* Pr_2_Me_2_), 21.31, 21.18 (Aryl─*C*H_3_), 10.16 (C─*C*H_3_ of I*^i^* Pr_2_Me_2_). **^1^H‐NMR** Data (benzene‐d_6_, TMS, ppm): 6.53 (s, 4H, Mes─*H*), 5.81 (b, 2H, N─C*H*CH_3_ of I*^i^* Pr_2_Me_2_), 2.52 (s, 12H, Mes─C*H*_3_), 2.15 (s, 6H, Mes─C*H*_3_), 1.62 (s, 6H, NC─C*H*_3_ of I*^i^* Pr_2_Me_2_), 1.30 (d, J = 6.8 Hz, 12H, NC─C*H*_3_ of I*^i^* Pr_2_Me_2_). **UV/Vis**: *λ* [nm] (*ε* [L mol^−1^ cm^−1^]) = 430 (2649), 323 (5393). **IR** (neat): *ν*(C = O) 1605, 1638, 1655.

**Analytical data for 4d**: **Yield**: 162 mg (0.62 mmol; 92%) of analytically pure **4d** as white crystalline solid. The analytical data are identical to those published.^[^ ^39^ ^]^

**Synthesis of 5a**: 100 mg of **4a** (0.20 mmol; 1.00 eq.) and 81 mg of Fe_2_(CO)_9_ (0.22 mmol; 1.10 eq.) were combined in a flask, 10 mL benzene was added and stirred for 3 hours. Subsequently, the solvent was removed and the product **5a** was isolated after recrystallization in toluene:THF 3:2 at −30 °C as a yellow crystalline solid. **Yield**: 103 mg (0.16 mmol; 77%) of analytically pure **5a** as yellow crystalline solid.

**mp**: 161–163 °C. **Anal. Calc**. (%) for C_31_H_34_FeGeN_2_O_6_: C, 56.49; 5.20 H, Found: C, 56.55; H, 5.41. **^13^C‐NMR** Data (THF‐d_8_, TMS, ppm): 242.31 (*C *= O), 217.10 (Fe(*C*O)_4_), 156.35 (N─*C*─N of IMe_4_), 143.20, 139.28, 134.46, 129.43 (Mes─*C*), 129.31 (N─*C*CH_3_ of IMe_4_), 35.52 (N─*C*H_3_ of IMe_4_), 20.92, 20.14 (Aryl─*C*H_3_), 8.54 (NC─*C*H_3_ of IMe_4_).**^1^H‐NMR** Data (THF‐d_8_, TMS, ppm): 6.74 (s, 4H, Mes─*H*), 3.76 (s, 6H, N─C*H*_3_ of IMe_4_), 2.23 (s, 6H, Mes─C*H*_3_), 2.22 (s, 6H, NC─C*H*_3_ of IMe_4_), 2.11 (s, 12H, Mes─C*H*_3_). **UV/Vis**: *λ* [nm] (*ε* [L mol^−1^ cm^−1^]) = 394 (670). **IR** (neat): *ν*(C = O) = 1631, 1604, *ν*(C≡O) = 1934, 1832.

**Synthesis of 5b**: 100 mg of **4b** (0.18 mmol; 1.00 eq.) and 73 mg of Fe_2_(CO)_9_ (0.20 mmol; 1.10 eq.) were combined in a flask, 10 mL benzene was added and stirred for 3 hours. Subsequently, the solvent was removed and the product **5b** was isolated after recrystallization in toluene:THF 3:2 at −30 °C as a yellow crystalline solid. **Yield**: 109 mg (0.15 mmol; 83%) of analytically pure **5b** as yellow crystalline solid.

**mp**: 157–159 °C. **Anal. Calc**. (%) for C_35_H_42_FeGeN_2_O_6_: C, 58.78; 5.92 H, Found: C, 58.55; H, 5.74. **^13^C‐NMR** Data (benzene‐d_6_, TMS, ppm): 241.48 (*C *= O), 216.96 (Fe*C*O) 158.03 (N─*C*─N of I*^i^* Pr_2_Me_2_), 142.85, 139.22, 134.80, 129.67 (Mes─*C*), 53.06 (N─*C*HCH_3_ of I*^i^* Pr_2_Me_2_), 21.53 (NC─*C*H_3_ of I*^i^* Pr_2_Me_2_), 21.03, 20.93 (Aryl─*C*H_3_), 10.27 (NC─*C*H_3_ of I*^i^* Pr_2_Me_2_). **^1^H‐NMR** Data (benzene‐d_6_, TMS, ppm): 6.68 (s, 4H, Mes─*H*), 5.85 (s broad, 2H, N─C*H*CH_3_ of I*^i^* Pr_2_Me_2_), 2.39 (s, 12H, Mes─C*H*_3_), 2.05 (s, 6H, Mes─C*H*_3_), 1.45 (s, 6H, NC─C*H*_3_ of I*^i^* Pr_2_Me_2_), 1.25 (d, J = 6.8 Hz, 12H, NC─C*H*_3_ of I*^i^* Pr_2_Me_2_). **UV/Vis**: *λ* [nm] (ε [L mol^−1^ cm^−1^]) = 410 (427), 389 (467). **IR** (neat): *ν*(C = O) = 1629, 1604, *ν*(C≡O) = 1930, 1910, 1890.

**Synthesis of 6a**: 100 mg of **4a** (0.20 mmol; 1.00 eq.) was dissolved in 10 mL THF and added to 21 µL Me_2_S·BH_3_ (0.22 mmol; 1.1 eq.; 0.801 g/mL) in 5 mL THF at 0 °C and slowly brought to room temperature. After stirring for 1 hour the solvent was removed and the product **6a** was isolated after washing the residue with Et_2_O as a yellow crystalline solid. **Yield**: 83 mg (0.16 mmol; 81%) of analytically pure **6a** as yellow crystalline solid.

**mp**: 177–179 °C. **Anal. Calc**. (%) for C_27_H_37_BGeN_2_O_2_: C, 64.21; 7.38 H, Found: C, 64.43; H, 7.45. **^13^C‐NMR** Data (benzene‐d_6_, TMS, ppm): 249.25 (*C *= O), 156.95 (N─*C*─N of IMe_4_), 144.99, 137.99, 132.89, 129.14, 127.96 (Mes─*C*), 127.33 (N─*C*CH_3_ of IMe_4_), 35.12 (N─*C*H_3_ of IMe_4_), 21.14, 19.85 (Aryl─*C*H_3_), 8.02 (NC─*C*H_3_ of IMe_4_). **^11^B‐NMR** (benzene‐d_6_, Et_2_O·BF_3_, ppm): −35.61 (─*B*H_3_). **^1^H‐NMR** Data (benzene‐d_6_, TMS, ppm): 6.67 (s, 4H, Mes─*H*), 3.53 (s, 6H, N─C*H*_3_ of IMe_4_), 2.37 (s, 12H, Mes─C*H*_3_), 2.09 (s, 6H, Mes─C*H*_3_), 1.05 (s, 6H, NC─C*H*_3_ of IMe_4_). **UV/Vis**: *λ* [nm] (*ε* [L mol^−1^ cm^−1^]) = 397 (415), 386 (394). **IR** (neat): *ν*(C = O) = 1635, 1610. *ν*(B─H) = 2370, 2280, 2210.

**Synthesis of 6b**: 100 mg of **4b** (0.18 mmol; 1.00 eq.)was dissolved in 10 mL THF and added to 19 µL Me_2_S·BH_3_ (0.20 mmol; 1.1 eq.; 0.801 g/mL) in 5 mL THF at 0 °C and slowly brought to room temperature. After stirring for 1 hour the solvent was removed and the product **6b** was isolated after washing the residue with Et_2_O as a yellow crystalline solid. **Yield**: 86 mg (0.15 mmol; 84%) of analytically pure **6b** as yellow crystalline solid.

**mp**: 157–159 °C. **Anal. Calc**. (%) for C_31_H_45_BGeN_2_O_2_: C, 66.35; 8.08 H, Found: C, 66.56; H, 8.24. **^13^C‐NMR** Data (benzene‐d_6_, TMS, ppm): 248.83 (*C *= O), 157.41 (N─*C*─N of I*^i^* Pr_2_Me_2_), 145.06, 137.78, 132.76, 129.05 (Mes─*C*), 128.15 (N─*C*CH_3_ of I*^i^* Pr_2_Me_2_), 52.61 (N─*C*HCH_3_ of I*^i^* Pr_2_Me_2_), 19.85 (NC─*C*H_3_ of I*^i^* Pr_2_Me_2_), 21.72, 21.14 (Aryl─*C*H_3_), 10.16 (NC─*C*H_3_ of I*^i^* Pr_2_Me_2_). **^11^B‐NMR** (benzene‐d_6_, Et_2_O·BF_3_, ppm): −34.09 (─*B*H_3_). **^1^H‐NMR** Data (benzene‐d_6_, TMS, ppm): 6.68 (s, 4H, Mes─*H*), 6.11 (sep, J = 6.8 Hz, 2H, N─C*H*CH_3_ of I*^i^* Pr_2_Me_2_), 2.36 (s, 12H, Mes─C*H*_3_), 2.08 (s, 6H, Mes─C*H*_3_), 1.51 (s, 6H, NC─C*H*_3_ of I*^i^* Pr_2_Me_2_), 1.26 (d, J = 7.0 Hz, 12H, NC─C*H*_3_ of I*^i^* Pr_2_Me_2_).). **UV/Vis**: *λ* [nm] (*ε* [L mol^−1^ cm^−1^]) = 398 (464), 384 (397). **IR** (neat): *ν*(C = O) = 1634, 1626, 1609, ν(B─H) = 2367, 2325, 2284.

**Synthesis of 8**: 300 mg of **7** (0.67 mmol; 1.00 eq.) was added as a solid to 12 mL of HCl dissolved in Et_2_O (19.3 mmol; 28.60 eq.; 1.6 M) at −50 °C. The reaction solution was slowly brought to room temperature followed by solvent removal. After adding *n*‐pentane to the residue the solution was filtrated and the product **3** was isolated as a yellow oil after removal of all volatiles. **Yield**: 239 mg (0.65 mmol; 96%) of analytically pure **8** as yellow oil.

**Anal. Calc**. (%) for C_20_H_24_GeO_2_: C, 65.09; H, 6.56, Found: C, 65.23; H, 6.63. **^13^C‐NMR** (C_6_D_6_, TMS, ppm): 231.85 (*C *= O), 143.10, 139.58, 132.94, 129.44 (Mes─*C*), 21.05, 19.26 (Aryl─*C*H_3_). **^1^H‐NMR** (C_6_D_6_, TMS, ppm): 6.53 (s, 4H, Mes─*H*), 5.24 (s, 2H, Ge─*H*), 2.14 (s, 12H, Mes─C*H*_3_), 2.01 (s, 6H, Mes─C*H*_3_). **UV/Vis**: *λ* [nm] (*ε* [L mol^− 1^ cm^−1^]) = 399 (620), 382 (686), 364 (555). **IR** (neat): *ν*(C = O) = 1651, 1606, *ν*(Ge─H) = 2059.

**Synthesis of 9**: 5 mL of CCl_4_ (52.69 mmol; 39 eq.)was added to 500 mg of **8** (1.36 mmol; 1.00 eq.), refluxed, and conversion followed via NMR, after about 3 hours full conversion was observed. Then the solvent was removed and the product **9** isolated as a yellow solid. **Yield**: 569 mg (1.30 mmol; 96%) of analytically pure **9** as yellow crystalline solid.

**Mp**: 111–113 °C. **Anal. Calc**. (%) for C_20_H_22_Cl_2_GeO_2_: C, 54.85; H, 5.06, Found: C, 54.78; H, 5.13. **^13^C‐NMR** (C_6_D_6_, TMS, ppm): 222.14 (*C *= O), 141.22, 137.75, 133.97, 129.47 (Mes─*C*), 21.10, 19.43 (Aryl─*C*H_3_). **^1^H‐NMR** (C_6_D_6_, TMS, ppm): 6.49 (s, 4H, Mes─*H*), 2.23 (s, 12H, Mes─C*H*_3_), 1.94 (s, 6H, Mes─C*H*_3_). **UV/Vis**: *λ* [nm] (*ε* [L mol^−1^ cm^−1^]) = 393 (1155), 370 (1090). **IR** (neat): *ν*(C = O) = 1606, 1658, 1687.

**Alternative synthesis of 4a**: 100 mg of **7** (0.22 mmol; 1.00 eq.) was dissolved in 10 mL THF, cooled to −70 °C, and 103 mg of **9** (0.24 mmol; 1.05 eq.) dissolved in 5 mL THF were slowly added. The reaction solution was stirred for 30 minutes at −70 °C and 31 mg of IMe_4_ (0.25 mmol; 1.1 eq.) dissolved in 5 mL THF were added, after which the cooling bath was removed and stirred for an hour. Subsequently, the solvent was removed, the crude product resuspended in 20 mL toluene and filtrated using a syringe filter. Again, the solvent was removed in vacuum, the product recrystallized in DME at −30 °C and isolated. **Yield**: 93 mg (0.19 mmol; 84%) of analytically pure **4a** as orange crystalline solid.

**Alternative synthesis of 4b**: 100 mg of **7** (0.22 mmol; 1.00 eq.) was dissolved in 10 mL THF, cooled to −70 °C and 103 mg of **9** (0.24 mmol; 1.05 eq.) dissolved in 5 mL THF were slowly added. The reaction solution was stirred for 30 minutes at −70 °C and 45 mg of I*^i^* Pr_2_Me_2_ (0.25 mmol; 1.10 eq.) dissolved in 5 mL THF were added, after which the cooling bath was removed and stirred for an hour. Subsequently, the solvent was removed, the crude product resuspended in 20 mL toluene and filtrated using a syringe filter. Again, the solvent was removed in vacuum, the product recrystallized in DME at −30 °C and isolated. **Yield**: 107 mg (0.20 mmol; 87%) of analytically pure **4b** as orange crystalline solid.

## Supporting Information

The authors have cited additional references within the Supporting Information.

## Author Contributions

M. P. was responsible for experimental investigations, formal analysis, visualization, data presentation, and writing original draft (lead). R. C. F. collected the X‐ray data and solved the crystal structures. A. K. was responsible for the DFT‐calculations. M. H. was in charge for methodology and conceptualization, review and editing of the manuscript (lead), project administration and funding acquisition.

## Conflict of Interest

The authors declare no conflict of interest.

## Supporting information



Supporting Information

## Data Availability

The data that support the findings of this study are available in the supplementary material of this article. In addition, all underlying NMR, IR and UV/Vis data supporting this work are openly available via the TU Graz repository a https://doi.org/10.3217/77g2f‐rtz43.
