# Supplementary material for: N‐Heterocyclic Carbene Stabilized Bisacylgermylenes
Source: Chemistry. 2025 Jun 12;31(38):e202501707. doi: 10.1002/chem.202501707 (PMC12238921; doi:10.1002/chem.202501707)
Supplement: Supplementary file 1 — Supporting Information [file CHEM-31-e202501707-s001.doc]

Supporting Information
©Wiley-VCH 2021
69451 Weinheim, Germany

*N*-Heterocyclic Carbene Stabilized Bisacylgermylenes

Matthias Paris,^[a]^ Roland C. Fischer,^[a]^ Anne-Marie Kelterer,^[b]^ and Michael Haas*^[a^

[a] DI Matthias Paris, Prof. Roland C. Fischer and Dr. Michael Haas
Institute of Inorganic Chemistry, Graz University of Technology
Stremayrgasse 9/IV, 8010 Graz (Austria)
E-mail: michael.haas@tugraz.at

[b] Prof. Anne-Marie Kelterer,
Institute of Physical and Theoretical Chemistry, Graz University of Technology
Stremayrgasse 9/I, 8010 Graz (Austria)

**Abstract:** In this contribution, a metal-free synthetic approach towards isolable bisacylgermylenes, a novel class of germylenes, is described. Starting from tetra(2,4,6-trimethylbenzoyl)germane **1** and bromo-tris(2,4,6-trimethylbenzoyl)germane **3**, we demonstrate that simple treatment with NHCs leads to two distinct types of reactivity: acyl abstraction and germylene stabilization. Reaction of **1** with NHCs produces imidazolium-substituted germenolates **2a**,**b** via a hydrogen atom transfer (HAT) mechanism. In contrast, compound **3** undergoes stepwise substitution to afford isolable NHC-stabilized bisacylgermylenes **4a**,**b**, which were structurally characterized by NMR and single-crystal X-ray diffraction. The UV/Vis spectra of **4a**,**b** shows two absorption bands in the visible region of the light, which do not overlap and have different characters. Reactivity studies revealed its nucleophilic character and resulted in the formation of several germylene/iron and germylene/boron complexes. The removal of the NHC at room temperature with triphenylborane leads to a degradation, mainly based on the instability of the unstabilized bisacylgermylene. To determine its reactivity, an alternative synthetic strategy was developed based on the reaction of the geminal bisgermenolates **7** with the bischloro-bis(2,4,6-trimethylbenzoyl)-germane **9**. Trapping reactions with NHCs results in the formation of **4a**,**b** in near quantitative yields. This work expands the synthetic scope of main group low-valent chemistry and introduces germylenes with tunable reactivity.

DOI:

Table of Contents

[Table of Contents 2](#__RefHeading___Toc198028160)

[NMR Spectroscopy 3](#__RefHeading___Toc198028161)

[X-Ray Crystallography Crystallographic Table 32](#__RefHeading___Toc198028162)

[UV/VIS Spectroscopy 35](#__RefHeading___Toc198028163)

[UV/VIS Spectra of compounds **2a**,**b**; **5a**,**b**; **6a**,**b**; **8** and **9** 35](#__RefHeading___Toc198028164)

[DFT Calculations 38](#__RefHeading___Toc198028165)

[Computed UV Spectra for compound **4a**,**b** 38](#__RefHeading___Toc198028166)

[Discussion of the DFT results 39](#__RefHeading___Toc198028167)

NMR Spectroscopy

Figure S1: ^13^C-NMR spectrum of **2a** (benzene-d_6_ solution, vs ext. TMS, ppm)

Figure S2: ^1^H-NMR spectrum of **2a** (benzene-d_6_ solution, vs ext. TMS, ppm)

Figure S3: ^13^C-NMR spectrum of **2b** (benzene-d_6_ solution, vs ext. TMS, ppm)

Figure S4: ^1^H-NMR spectrum of **2b** (benzene-d_6_ solution, vs ext. TMS, ppm)

Figure S5: ^13^C-NMR spectrum of **4a** (benzene-d_6_ solution, vs ext. TMS, ppm)

Figure S6: ^1^H-NMR spectrum of **4a** (benzene-d_6_ solution, vs ext. TMS, ppm)

Figure S7: ^13^C-NMR spectrum of **4c** (CDCl_3_ solution, vs ext. TMS, ppm)

Figure S8: ^1^H-NMR spectrum of **4c**(CDCl_3_ solution, vs ext. TMS, ppm)

Figure S9: ^13^C-NMR spectrum of **4b** (benzene-d_6_ solution, vs ext. TMS, ppm)

Figure S10: ^1^H-NMR spectrum of **4b** (benzene-d_6_ solution, vs ext. TMS, ppm)

Figure S11: ^13^C-NMR spectrum of **4d** (CDCl_3_ solution, vs ext. TMS, ppm)

Figure S12: ^1^H-NMR spectrum of **4d** (CDCl_3_ solution, vs ext. TMS, ppm)

Figure S13: ^13^C-NMR spectrum of **5a** (THF-d_8_ solution, vs ext. TMS, ppm)

Figure S14: 1H-NMR spectrum of **5a** (THF-d_8_ solution, vs ext. TMS, ppm)

Figure S15: ^13^C-NMR spectrum of **5b** (benzene-d_6_ solution, vs ext. TMS, ppm)

Figure S16: ^1^H-NMR spectrum of **5b** (benzene-d_6_ solution, vs ext. TMS, ppm)

Figure S17: ^13^C-NMR spectrum of **6a** (benzene-d_6_ solution, vs ext. TMS, ppm)

Figure S18: ^11^B-NMR (decoupled) spectrum of **6a** (benzene-d_6_ solution, vs ext. TMS, ppm)

Figure S19: ^11^B-NMR (coupled) spectrum of **6a** (benzene-d_6_ solution, vs ext. TMS, ppm)

Figure S20: ^1^H-NMR spectrum of **6a** (benzene-d_6_ solution, vs ext. TMS, ppm)

Figure S21: ^13^C-NMR spectrum of **6b** (benzene-d_6_ solution, vs ext. TMS, ppm)

Figure S22: ^11^B-NMR (decoupled) spectrum of **6b** (benzene-d_6_ solution, vs ext. TMS, ppm)

Figure S23: ^11^B-NMR (coupled) spectrum of **6b** (benzene-d_6_ solution, vs ext. TMS, ppm)

Figure S24: ^1^H-NMR spectrum of **6b** (benzene-d_6_ solution, vs ext. TMS, ppm)

Figure S25: Crude ^13^C-NMR spectrum after the reaction of **4b** with BPH_3_ (benzene-d_6_ solution, vs ext. TMS, ppm)

Figure S26: ^13^C-NMR spectrum of **8** (benzene-d_6_ solution, vs ext. TMS, ppm)

Figure S27: ^1^H-NMR spectrum of **8** (benzene-d_6_ solution, vs ext. TMS, ppm)

Figure S28: ^13^C-NMR spectrum of **9** (benzene-d_6_ solution, vs ext. TMS, ppm)

Figure S29: ^1^H-NMR spectrum of **9** (benzene-d_6_ solution, vs ext. TMS, ppm)

0 min

60 min

120 min

X-Ray Crystallography Crystallographic Table

Table S1: Crystallographic data and details of measurements for compound **2b**, **4a**,**b, 5a**,**b**, **6a**, **8** and **9**. Mo Kα (λ=0.71073Å). R1= Σ/ |F_o_|- |F_c_|/|Σ|F_d_; wR2 = [Σ_w_(F_o_^2^-F_2_^2^)2/Σ_w_(F_o_^2^)^2^]^1/2^

| **Compound** | **2b** | **4a** | **4b** | **5a** | **5b** | **6a** | **8** | **9** |
| --- | --- | --- | --- | --- | --- | --- | --- | --- |
| CCDC No. | 2374269 | 2374270 | 2374271 | 2374272 | 2374273 | 2374274 | 2374275 | 2374276 |
| Formula | C_56_H_69_GeN_2_O_3_ | C_27_H_34_GeN_2_O_2_ | C_31_H_42_GeN_2_O_2_ | C_31_H_42_FeGeN_2_O_6_ | C_35_H_42_FeGeN_2_O_6_ | C_27_H_37_BGeN_2_O_2_ | C_20_H_24_GeO_2_ | C_20_H_22_Cl_2_GeO_2_ |
| M_r_ (g mol^-1^) | 890.72 | 491.17 | 586.31 | 659.10 | 715.14 | 504.98 | 368.98 | 437.86 |
| a (Å) | 8.904(5) | 28.1685(16) | 41.678(4) | 11.5156(6) | 10.1827(5) | 11.0468(5) | 21.421(3) | 13.9093(14) |
| b (Å) | 13.8257(8) | 11.1310(6) | 17.6542(17) | 16.9837(10) | 14.4346(9) | 24.4089(14) | 21.259(3) | 8.9014(8) |
| c (Å) | 20.4863(13) | 15.2879(9) | 8.4834(7) | 17.3972(10) | 23.1910(14) | 9.9534(6) | 16.298(2) | 16.1741(15) |
| α (°) | 78.705(4) | 90 | 90 | 90 | 90 | 90 | 90 | 90 |
| β (°) | 84.373(4) | 90 | 100.644(5) | 90 | 92.108(3) | 103.5460(10) | 100.633(7) | 97.627(4) |
| γ (°) | 85.736(4) | 90 | 90 | 90 | 90 | 90 | 90 | 90 |
| V (Å3) | 2457.4(3) | 4793.4(5) | 6134.6(10) | 3402.5(3) | 3406.4(3) | 2609.2(2) | 7294.5(17) | 1984.8(3) |
| Z | 2 | 4 | 8 | 4 | 4 | 4 | 16 | 4 |
| Crystal size (mm) | 0.22x0.17x0.12 | 0.16x0.13x0.10 | 0.26x0.09x0.07 | 0.22×0.17×0.15 | 0.18×0.15×0.11 | 0.11x0.09x0.07 | 0.16x0.13x0.08 | 0.19x0.07x0.07 |
| Crystal habit | block, orange | block, orange | block, orange | block, yellow | block, orange | block, yellow | plate, yellow | plate, yellow |
| Crystal system | triclinic | orthorhombic | monoclinic | orthorhombic | Monoclinic | monoclinic | monoclinic | monoclinic |
| Space group | P -1 | F d d 2 | C 1 2/c 1 | P2_1_2_1_2_1_ | P2_1_/n | C 1 c 1 | C 1 2 1 | C 1 2/c 1 |
| *d_calc_* (Mg m^-3^) | 1.204 | 1.361 | 1.270 | 1.427 | 1.394 | 1.286 | 1.344 | 1.465 |
| μ (mm^-1^) | 0.668 | 1.304 | 1.031 | 1.360 | 1.354 | 1.200 | 1.687 | 1.823 |
| T (K) | 100.12 | 99.99 | 99.31 | 99.95 | 99.95 | 296.15 | 99.15 | 99.97 |
| 2θ range (°) | 4.07-60.16 | 4.75-54.00 | 3.77-54.00 | 4.242-60.066 | 3.514-60.432 | 4.144-59.228 | 2.54-55.27 | 5.08-56.08 |
| F (000) | 950 | 2064.0 | 2488.0 | 1520.0 | 1488.0 | 1064.0 | 3072 | 896 |
| T_min_, T_max_ | 0.563, 0.746 | 0.348, 0.746 | 0.526, 0.746 | 0.754, 0.822 | 0.578, 0.746 | 0.640, 0.746 | 0.769, 0.874 | 0.746, 0.549 |
| R_int_ | 0.0953 | 0.1209 | 0.0734 | 0.0823 | 0.1096 | 0.0461 | 0.1268 | 0.0606 |
| No. of measured, independent and observed [I**]** > 2σ(I)] reflections | 89437, 14322, 10677 | 24067, 2592, 2170 | 90337, 6640, 5493 | 69175, 9778, 8800 | 162769, 10015, 7699 | 70143, 7291, 7074 | 132148, 16671, 12570 | 43516, 2386, 2128 |
| No. of parameters, restraints | 574, 0 | 197, 32 | 397, 36 | 426, 0 | 418, 0 | 317, 2 | 854, 61 | 118, 0 |
| Δ›max, Δ›min (e Å^-3^) | 0.75, -1.01 | 0.29, -0.38 | 0.85, -0.66 | 0.55, -0.57 | 0.76, -0.61 | 0.27, -0.21 | 1.79, -2.98 | 0.58, -0.69 |
| R1, wR2 (all data) | R1 = 0.0868  wR2 = 0.1025 | R1 = 0.0760.  wR2 = 0.1197. | R1 = 0.0715  wR2 = 0.1063 | R1 = 0.0377  wR2 =0.0734 | R1 = 0.0574  wR2 =0.0798 | R1 = 0.0204  wR2 = 0.0504 | R1 = 0.1110  wR2 = 0.1722 | R1 = 0.0389  wR2 = 0.0821 |
| R1, wR2 (>2σ) | R1 = 0.0528  wR2 = 0.0938 | R1 = 0.0549.  wR2 = 0.1126. | R1 = 0.0522  wR2 = 0.0995 | R1 = 0.0302  wR2 = 0.0710 | R1 = 0.0340  wR2 = 0.0725 | R1 = 0.0196  wR2 = 0.0502 | R1 = 0.0783  wR2 = 0.1561 | R1 = 0.0309  wR2 = 0.0776 |


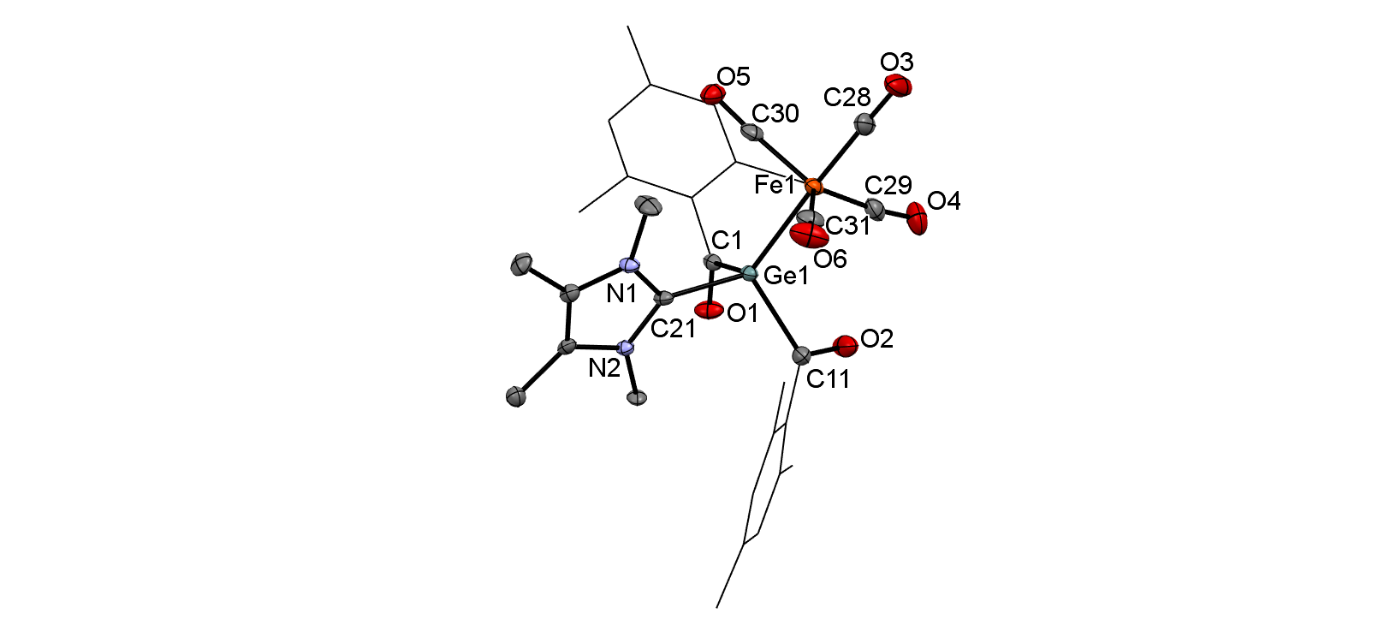


**Figure S30**: ORTEP representation for compound **5a**. Thermal ellipsoids are depicted at the 50% probability level. Hydrogen atoms are omitted and mesityl groups are displayed as wireframes for clarity. Selected bond lengths (Å) and bond angles (deg) with estimated standard deviations: ΣαGe(1) 301.97, Ge(1)-C(1) 2.057(2), Ge(1)-C(11) 2.055(2), Ge(1)-C(21) 2.041(2), Ge(1)-Fe(1) 2.3879(4), C(21)-N(1) 1.360(3), C(21)-N(2) 1.349(3), C(1)-O(1) 1.227(3), C(11)-O(2) 1.206(3), Fe(1)-C(28) 1.780(3), Fe(1)-C(29) 1.800(3), Fe(1)-C(30) 1.784(3), Fe(1)-C(31) 1.781(3), C(28)-O(3) 1.146(3), C(29)-O(4) 1.151(3), C(30)-O(5) 1.161(3), C(31)-O(6) 1.156(3).


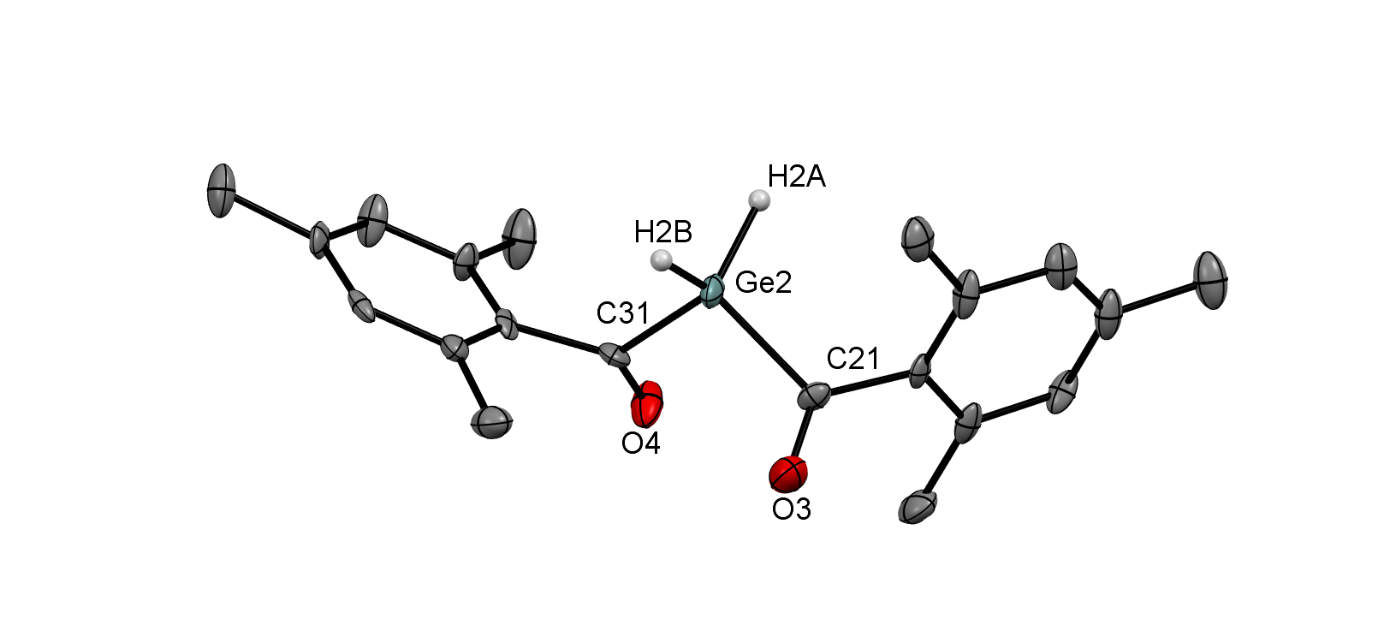


**Figure S31**: ORTEP representation for compound **8**. Thermal ellipsoids are depicted at the 50% probability level. Hydrogen atoms are mostly omitted for clarity. Selected bond lengths (Å) and bond angles (deg) with estimated standard deviations: C(21)-Ge(2)-C(31) 104.9(4), Ge(2)-H(2A) 1.5200, Ge(2)-H(2B) 1.5200, Ge(2)-C(21) 2.010(10), Ge(2)-C(31) 2.026(11), C(21)-O(3) 1.181(13), C(31)-O(4) 1.201(14).


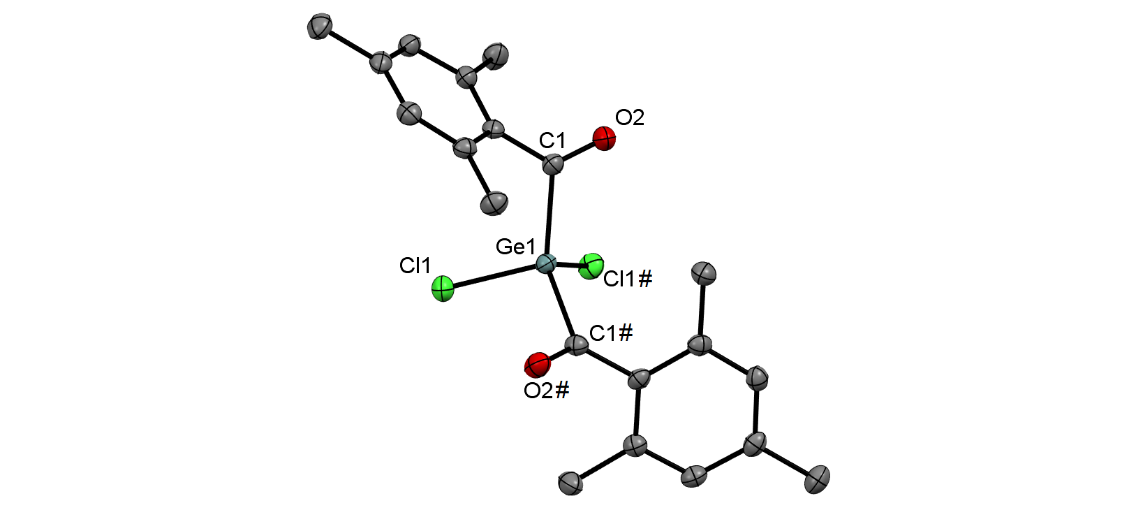


**Figure S32:** ORTEP representation for compound **9**. Thermal ellipsoids are depicted at the 50% probability level. Hydrogen atoms are omitted for clarity. Selected bond lengths (Å) and bond angles (deg) with estimated standard deviations: ΣαGe(1) 324.40, C(1)-Ge(1)-C(1)# 131.47(11), Ge(1)-Cl(1) 2.1560(5), Ge(1)-C(1) 2.038(2), C(1)-O(2) 1.202(3).

UV/VIS Spectroscopy

UV/VIS Spectra of compounds 2a,b; 5a,b; 6a,b; 8 and 9


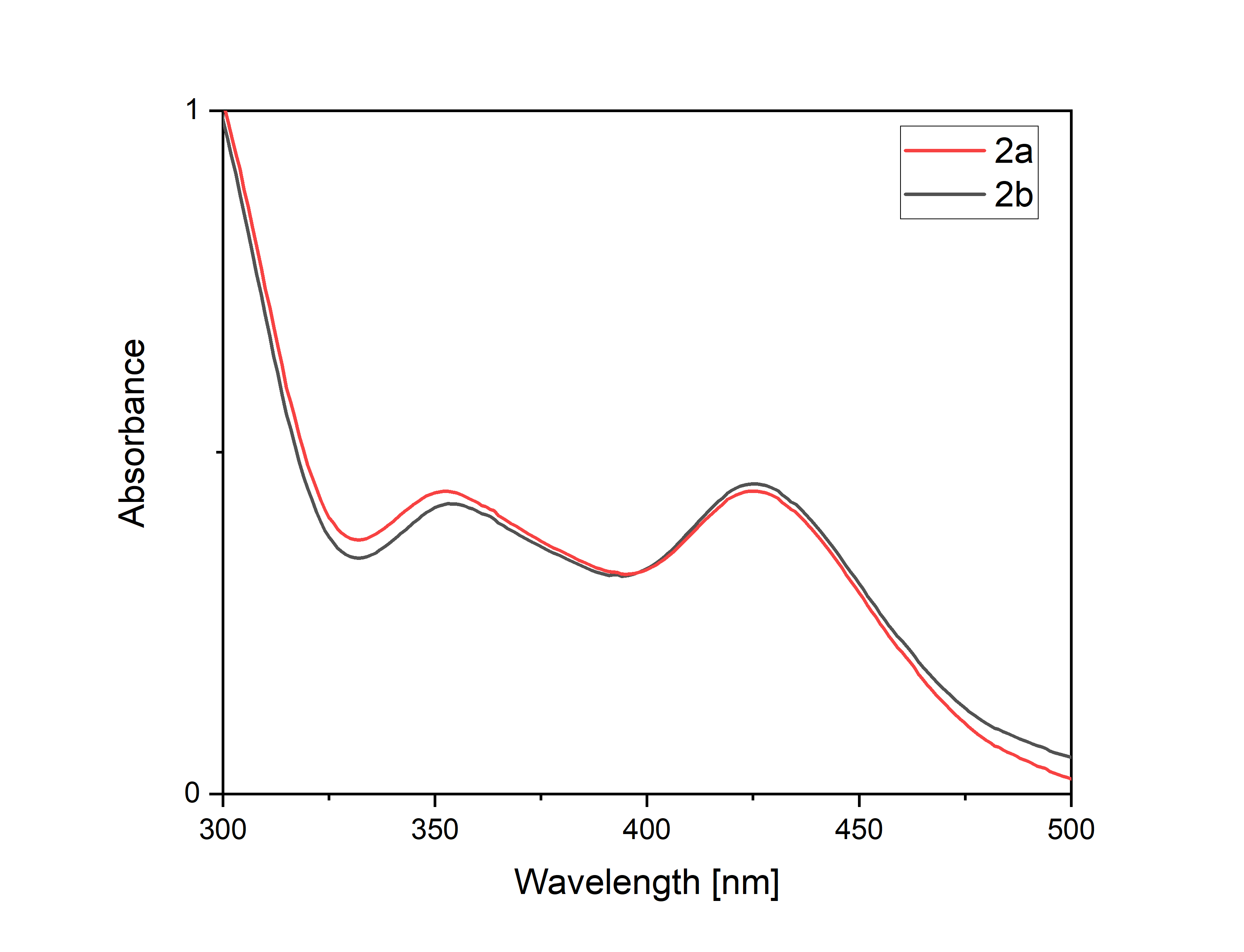


**Figure S33.** UV/Vis spectra of **2a**,**b** in THF at 1• 10^-4^mol/L.


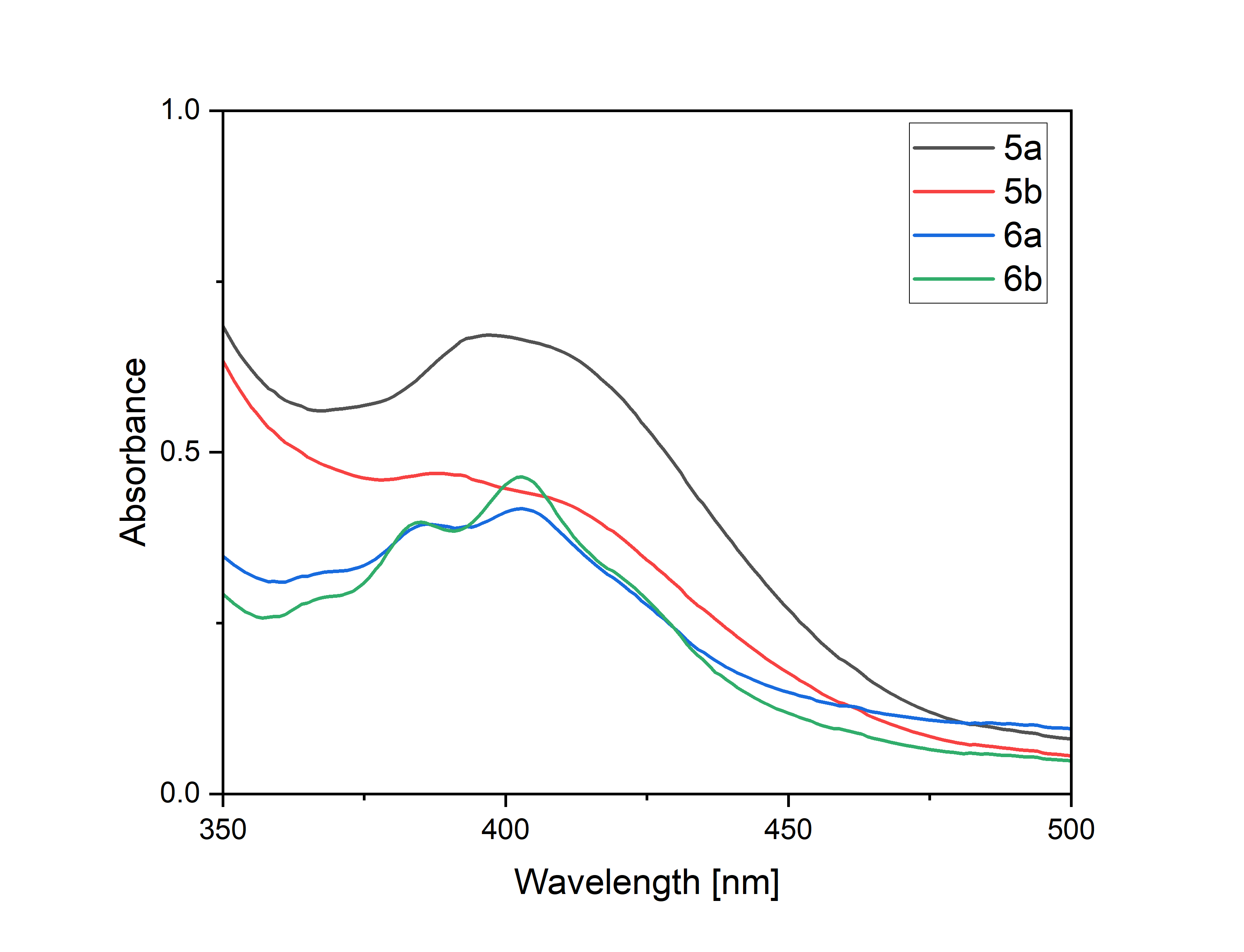


**Figure S34.** UV/Vis spectra of **5a**,**b** and **6a,b** in THF at 1• 10^-3^mol/L.


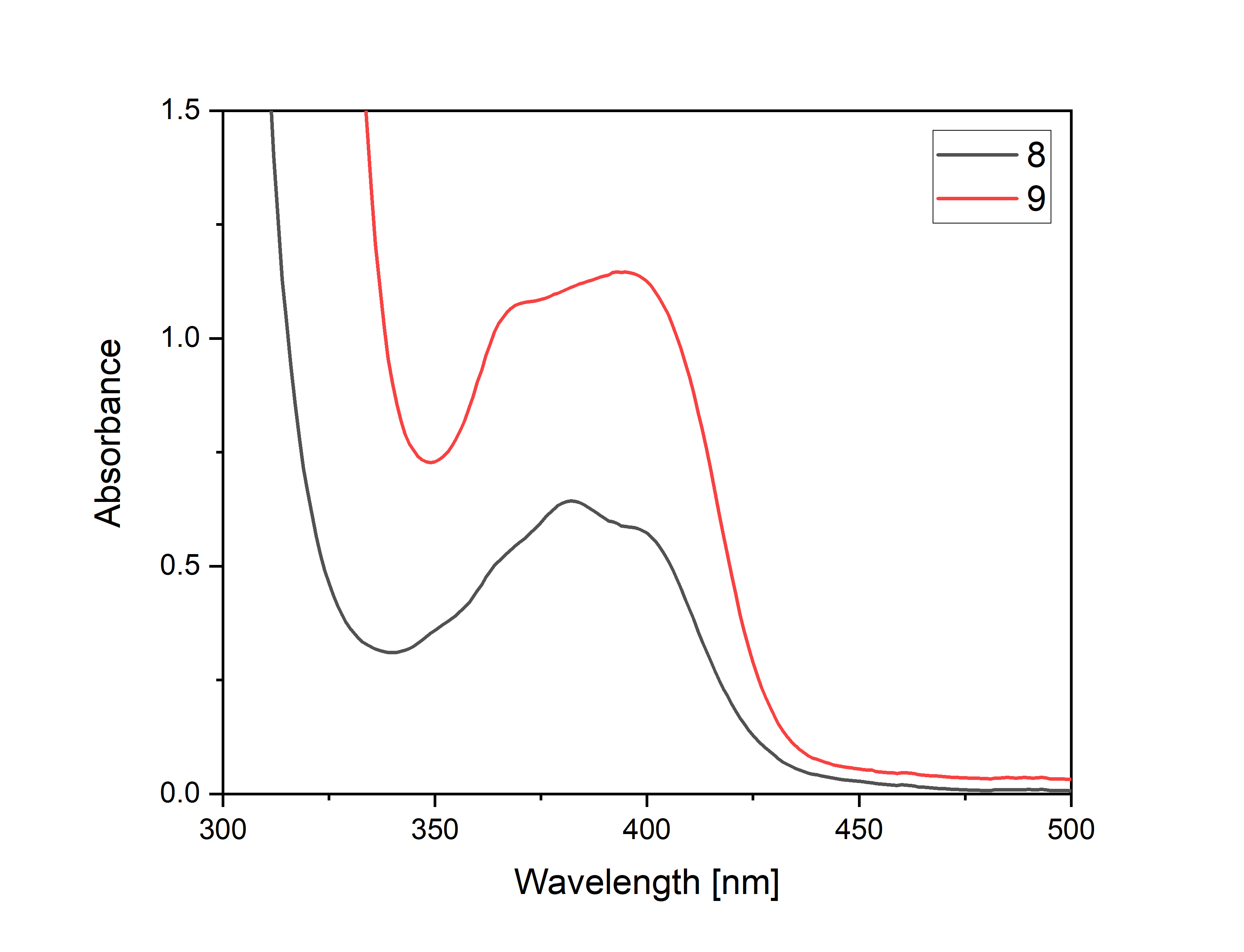


**Figure S35.** UV/Vis spectra of **8** and **9** in THF at 1• 10^-3^mol/L.

DFT Calculations

Computed UV Spectra for compound 4a,b

**Table S2. Absorption data of compound 4a, computed with TDDFT using the PBEh-3c method: wavelength (in nm), vertical excitation energy (in eV), oscillator strength f and MO contributions (c^2^ of the MO coefficients c).**

| **excited state** | **λ/nm** | **E/eV** | **f** | **orbital contributions (c^2^)** |
| --- | --- | --- | --- | --- |
| S_1_ | 390.2 | 3.178 | 0.0433 | H→L (0.42), H-1→L (0.22) |
| S_2_ | 372.0 | 3.333 | 0.0598 | H→L+1 (0.44), H-1→L+1 (0.15), H→L (0.10) |
| S_3_ | 310.8 | 3.990 | 0.0384 | H-1→L (0.38), H→L (0.30) |
| S4 | 307.5 | 4.032 | 0.0403 | H→L+3 (0.42), H→L+1 (0.22) |
| S_5_ | 277.1 | 4.475 | 0.1245 | H→L+3 (0.33), H-1→L+1 (0.16), H→L+1 (0.14) |
| S_6_ | 263.6 | 4.703 | 0.0119 | H→L+2 (0.54), H→L+5 (0.25) |

**Table S3.** Geometry and relevant orbitals of compound **4a**, computed with the PBEh-3c method. Contour values of the orbitals are 0.04 a.u.

| 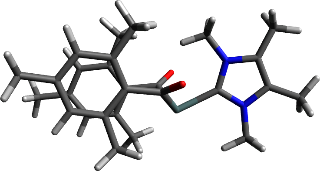 | 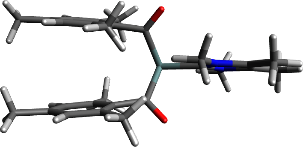 | 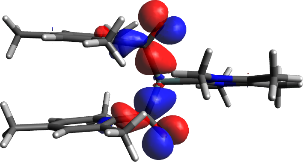 | 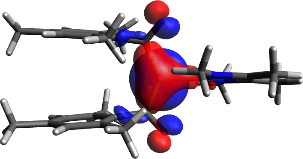 |
| --- | --- | --- | --- |
| geometry (rotated) | geometry | HOMO-1 | HOMO |
| 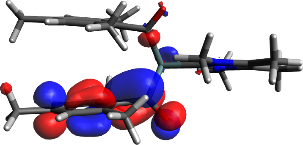 | 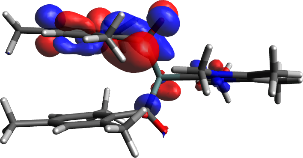 | 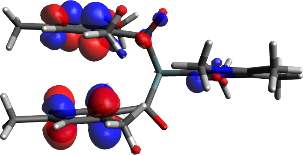 | 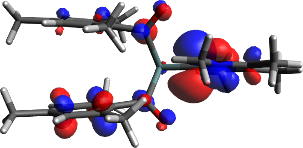 |
| LUMO | LUMO+1 | LUMO+2 | LUMO+3 |

**Table S4.** Calculated absorption data of compound **4b**, computed with TDDFT using the PBEh-3c method: wavelength (in nm), vertical excitation energy (in eV), oscillator strength f and MO contributions (c^2^ of the MO coefficients c).

| **excited state** | **λ/nm** | **E/eV** | **f** | **orbital contributions (c^2^)** |
| --- | --- | --- | --- | --- |
| S_1_ | 392.0 | 3.163 | 0.0630 | H→L (0.55), H-1→L (0.19) |
| S_2_ | 360.2 | 3.442 | 0.0652 | H→L+1(0.43), H-1→L+1(0.15) |
| S_3_ | 316.8 | 3.913 | 0.0227 | H→L+3(0.43), H→L+1 (0.20), H→L+2(0.16) |
| S_4_ | 312.3 | 3.970 | 0.0514 | H-1→L(0.41), H→L (0.24), H→L+1(0.17) |
| S_5_ | 276.6 | 4.482 | 0.0367 | H→L+3(0.36), H→L+2(0.20), H→L+4(0.15), H→L+1(0.11) |
| S_6_ | 274.3 | 4.520 | 0.1144 | H→L+2(0.43), H-1→ L+1 (0.22) |

**Table S5.** Geometry and relevant orbitals of compound **4b**, computed with the PBEh-3c method. Contour values of the orbitals are 0.04 a.u.

| 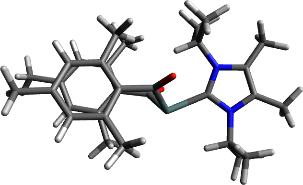 | 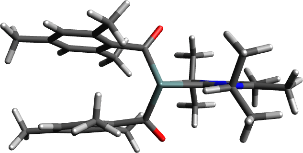 | 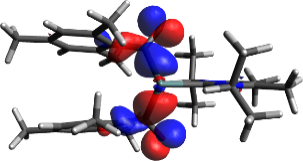 | 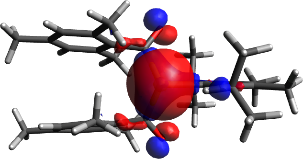 |
| --- | --- | --- | --- |
| geometry (rotated) | geometry | HOMO-1 | HOMO |
| 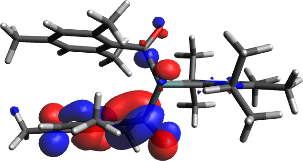 | 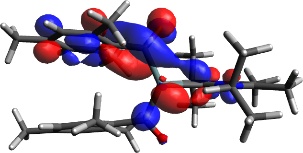 | 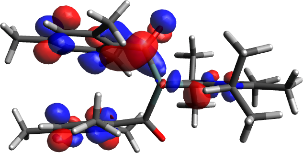 | 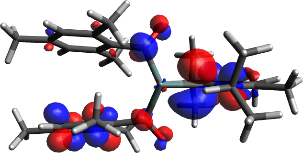 |
| LUMO | LUMO+1 | LUMO+2 | LUMO+3 |
|  |  |  |  |

Discussion of the DFT results

**4a** shows two main bands with different character: the band at ca. 430 nm comprises of the n-π* excitation from HOMO-1 and HOMO located at the n orbital of the carbonyl group and Germanium, respectively, to LUMO and LUMO+1 located at the mesitoyl moieties. The band at ca. 320 nm has a shoulder at 350 nm including some charge-transfer character involving excitations to the π-system of the NHC moiety.

**4b** conforms with the same type of excitations, but with a slight red-shift of the second band.


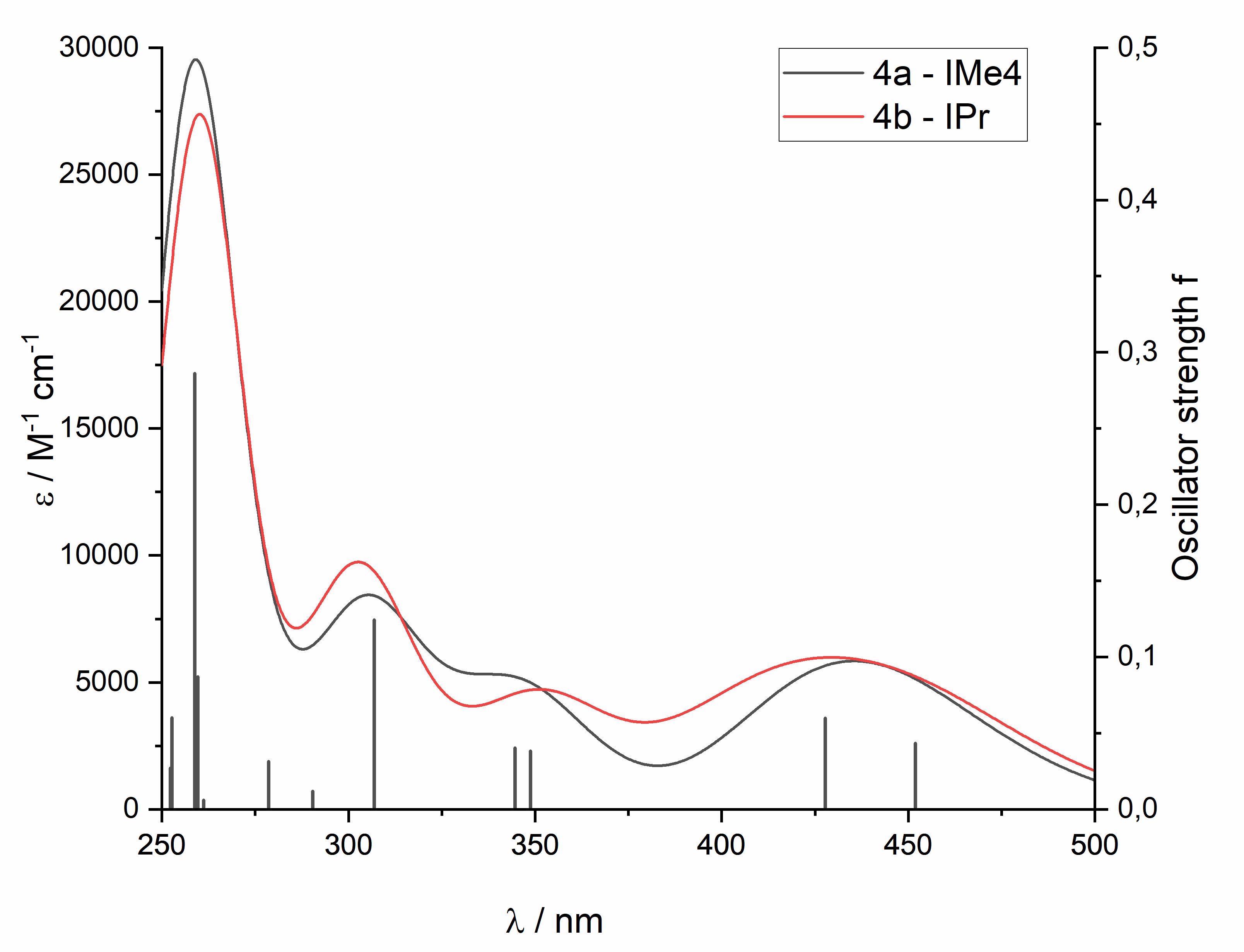


**Figure S36.** Simulated PBEh-3c UV/Vis absorption spectrum of **4a** (black) and **4b** (red) with vertical excitations of **4a** (black vertical lines). The bands are red-shifted by 3500cm^-1^ to show agreement with the experimental first band position.
